# Dietary nitrate and nitrite protect against doxorubicin‐induced cardiac fibrosis and oxidative protein damage in tumor‐bearing mice

**DOI:** 10.1002/2211-5463.70139

**Published:** 2025-11-20

**Authors:** Rama D. Yammani, Xiaofei Chen, Nildris Cruz‐Diaz, Xuewei Zhu, Swati Basu, Daniel B. Kim‐Shapiro, David R. Soto‐Pantoja, Leslie B. Poole

**Affiliations:** ^1^ Department of Biochemistry Wake Forest University School of Medicine Winston‐Salem NC USA; ^2^ Department of Internal Medicine, Section on Molecular Medicine Wake Forest University School of Medicine Winston‐Salem NC USA; ^3^ Department of Surgery Wake Forest University School of Medicine Winston‐Salem NC USA; ^4^ Center for Redox Biology and Medicine Wake Forest University School of Medicine Winston‐Salem NC USA; ^5^ Department of Physics Wake Forest University Winston‐Salem NC USA; ^6^ Cardiovascular Sciences Center Wake Forest University School of Medicine Winston‐Salem NC USA; ^7^ Department of Cancer Biology Wake Forest University School of Medicine Winston‐Salem NC USA; ^8^ Comprehensive Cancer Center Wake Forest University School of Medicine Winston‐Salem NC USA

**Keywords:** cardioprotection, cardiotoxicity, chemotherapy, nitrate, nitric oxide, nitrite

## Abstract

Anthracycline‐induced cardiotoxicity remains a major limitation in cancer therapy, affecting long‐term cardiovascular health in survivors. Dietary nitrate supplementation has shown cardioprotective effects in preclinical models of doxorubicin (Dox)‐induced and ischemia–reperfusion injury, but it is unclear whether nitrate and/or nitrite (NOx) would have adverse effects on the anticancer efficacy of the drug. To evaluate Dox efficacy against triple‐negative breast cancer (TNBC) in the presence of dietary nitrate and nitrite, tumor‐bearing BALB/c mice (*N* = 5 mice per group, 10 mice total) were treated with four weekly intravenous doses of Dox with or without NOx supplementation of their drinking water. Cardiac tissue from the NOx‐treated mice exhibited less fibrosis and lower levels of 4‐hydroxynonenal‐modified proteins, a marker of lipid oxidation and oxidative stress. Tumor sizes varied, but most regressed by the final Dox dose. Importantly, NOx supplementation did not compromise the antitumor efficacy of Dox nor did it promote pulmonary metastasis; instead, a trend toward fewer metastatic lesions was observed. These findings support the potential clinical use of dietary nitrate and nitrite as adjuncts to Dox treatment to mitigate cardiotoxicity without impairing anticancer outcomes.

## Graphical abstract

Chemotherapies such as doxorubicin can have toxic effects on healthy cardiovascular/heart tissue. Following up on a doxorubicin toxicity study in mice without tumors where nitrate water was cardioprotective (lessened toxicity), this study with tumor‐bearing mice undergoing doxorubicin treatment showed no negative effect of nitrate and nitrite on drug potency, no increase in metastasis to lung, but retention of cardioprotective effects.

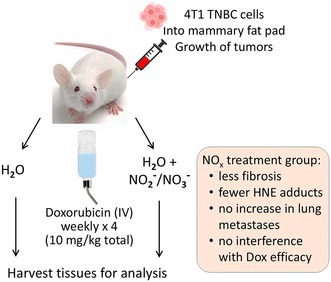

**Abbreviations**
ACE: angiotensin‐converting enzyme
Dox: doxorubicin
ECHO: echocardiography
EF: ejection fraction
eNOS: endothelial nitric oxide synthase
FS: fractional shortening
GAPDH: glyceraldehyde‐3‐phosphate dehydrogenase
H&E: hematoxylin and eosin
HNE: 4‐hydroxynonenal
I/R: ischemia–reperfusion
LV: left ventricle
NOx: nitrate and/or nitrite
Prx3: peroxiredoxin‐3
RNS: reactive nitrogen species
ROS: reactive oxygen species
SEM: standard error of the mean
TNBC: triple‐negative breast cancer
Topo2B: topoisomerase 2B

Cardiotoxicity as a result of anthracycline chemotherapy is a significant health concern for large populations of survivors. Anthracycline administration is widespread as these are potent drugs employed, for example, against especially hard‐to‐treat, triple‐negative breast cancer (TNBC) for which targeted therapies are ineffective. Unfortunately, their off‐target toxic effects on heart function, both acute and chronic, are significant and dose‐limiting. The urgency of this problem is driving extensive research in this area.

A number of cardioprotective co‐therapies being tested focus on oxidative stress and redox‐mediated protection, as anthracyclines such as doxorubicin (Dox) generate reactive oxygen species (ROS), including peroxynitrite, particularly in mitochondria [1, 2]. To date, one cardioprotective therapy, an iron chelator (dexrazoxane), is approved for clinical use, but is rarely recommended due to problematic side effects [2]. Potential new therapies continue to be tested, including dietary nitrate and hydrogen sulfide, which are cardioprotective in rodent models [3, 4], but much more research is needed to validate these new therapies and understand both the toxic and protective mechanisms involved.

One approach, as developed recently by members of our research group, is to generate ROS‐triggered prodrugs that release both the anthracycline (e.g., doxorubicin, or Dox) and hydrogen sulfide, with the goal of maintaining or enhancing Dox efficacy against the tumor while protecting the heart and vasculature from drug‐mediated damage [5], but this awaits further testing. Another approach focuses on dietary nitrate provided before and/or during Dox administration, as ingested nitrate is well‐established to be actively taken up by the salivary glands and provide positive effects (protection from ischemia–reperfusion (I/R) injury to the heart, and enhancement of exercise efficacy) over sustained periods [3, 6, 7, 8, 9]. Although molecular mechanisms of cardiac and vascular protection by dietary nitrate are not fully understood, multiple nitrogen oxide species (notably nitrite, ${\mathrm{NO}}_{2}^{-}$; nitric oxide, **^·^** NO; and nitroxyl, HNO) are likely to play a role in these effects. Oral bacteria and other factors bioactivate dietary nitrate present in saliva to generate nitrite and ^·^NO in blood and tissues, particularly under hypoxic conditions [6, 10]. Inhalation of sodium nitrite directly by patients has itself been shown to be of therapeutic value in combatting pulmonary arterial hypertension [11].

One study with mice challenged acutely with high‐dose Dox demonstrated the efficacy of dietary nitrate in cardioprotection as shown by lowered cardiac fibrosis and lipid oxidation [3]. Still, to our knowledge, tumor‐bearing animal models have not been tested. In the acute study, mice were sacrificed 1 week after challenge with a single high dose of Dox (15 mg·kg^−1^), testing whether sodium nitrate in the drinking water of adult male CF‐1 mice was cardioprotective. Data obtained showed that the nitrate significantly decreased the Dox‐induced impairment of ventricular function, decreased tissue lipid peroxidation, and protected against altered levels of specific antioxidant proteins in mitochondria (shown in a subsequent paper conducting proteomics analyses on these samples) [12]. Mechanisms through which the nitrate is cardioprotective are only poorly defined, but proteomics of cardiac tissue indicated significant increases, with Dox exposure, in the level of the mitochondrial peroxiredoxin protein, Prx3, which was not increased in the nitrate‐treated animals [12]; protection of cardiomyoblasts by hydrogen sulfide *in vitro* was also linked to similar changes in Prx3 levels [13], although it is not clear how keeping Prx3 levels low would offer a benefit against Dox toxicity.

Acute models of the cardiotoxic effects of chemotherapies have utilized large doses and tumor‐free animals, neither of which adequately reflects human exposures. With this study, we specifically sought evidence for or against the hypothesis that the nitrate‐Dox combination would maintain anticancer efficacy and perhaps even sensitize TNBC tumors treated with Dox. While animal numbers were limited to 5 mice per group for the two groups, with and without NOx supplementation, this key question needed to be addressed before moving forward with larger preclinical studies. Analyses of the tumor sizes and tissue samples from the Dox‐treated mice, with or without nitrate and nitrite (NOx) supplementation in their drinking water, revealed that NOx‐treated animals exhibited reduced cardiac fibrosis and lower levels of lipid peroxidation in heart tissues. NOx supplementation did not impair the antitumor efficacy of Dox in inducing tumor regression, nor did it increase tumor metastasis to lung. In sum, these results suggest that there would be significant value in pursuing more in‐depth studies to evaluate the therapeutic values of NOx supplementation using additional animal models and human subjects.

## Materials and methods

### Experimental design and treatment protocol

As noted, the treatments and interventions were focused on assessing effects of dietary NOx on Dox‐treated mice with established breast cancer tumors for the first time. The primary goal was to conduct a pretest to ensure that, in a tumor‐bearing mouse model, observed cardioprotective effects of dietary NOx were not accompanied by a detectable decrease in the antitumor Dox efficacy. Inclusion of a sham‐treated group (administered saline instead of Dox) was considered, but decided against, as we recognized that the tumors would grow too large over this time course and the animals would have reached their humane endpoint, or would have had to undergo surgery to have their tumors resected in order to survive longer as in a similar earlier study by members of this group [14]. Either way, this would have contributed little to the primary goals of our study.

The tumor‐bearing mouse model used female BALB/c mice and syngeneic 4T1 triple‐negative breast cancer (TNBC) cells obtained from the American Type Culture Collection. Prior to injection, the 4T1 were cultured in DMEM supplemented with 10% fetal bovine serum with penicillin, streptomycin, and glutamine in an incubator kept at 37 °C and 5% CO_2_. Cell line authentication was performed by next‐generation sequencing, and mycoplasma testing confirming these cells to be mycoplasma‐free was performed by the Cell Engineering Shared Resource at Wake Forest University School of Medicine.

**Fig. 1:**
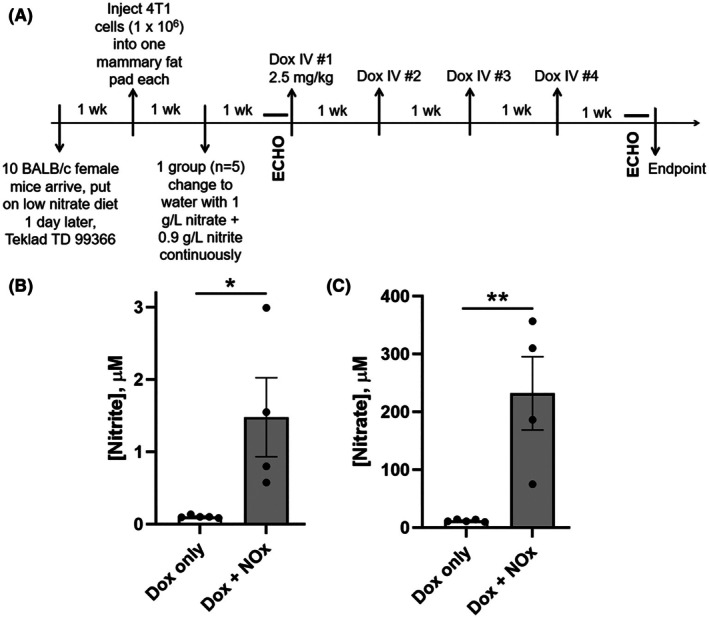
Experimental plan for assessing NOx (nitrate plus nitrite) supplementation effects on Dox‐treated mice bearing triple‐negative breast cancer (TNBC) tumors, and associated systemic nitrate and nitrite levels in plasma. (A) Following injection of 4T1 cancer cells into BALB/c mice, switching half of the animals after 1 week to drinking water supplemented with sodium nitrate and sodium nitrite, and growth of tumors for 13 days, echocardiography (ECHO) was performed, then mice were treated with four weekly doses of Dox, again evaluated by ECHO, then sacrificed to harvest tissues for analysis. (B and C) Plasma was evaluated for concentrations of nitrite (B) and nitrate (C). Data are presented as the mean ± standard error of the mean (SEM), with *n* = 5 and *n* = 4 for Dox only and Dox + NOx‐treated groups, respectively (see text and Fig. 2 legend for details). Statistical analysis in (B) and (C) was conducted by two‐tailed Student's *t*‐test. *, *P* < 0.05, **, *P* < 0.01.

Female 6‐week‐old BALB/c mice (*n* = 10) were purchased from Jackson Laboratory (Bar Harbor, ME, USA) and put on a low nitrate diet (Harlan Teklad TD 99366, Madison, WI, USA) 1 day post arrival. This chow was noted in an earlier study to result in about half the level of nitrite and nitrate in plasma of mice (0.2 μm nitrite) as the standard NIH‐31 chow (at ~ 0.4 μm nitrite), and could correspond to dietary NOx intake for some human patients [15]. One week later mice were each injected with the syngeneic 4T1 TNBC cells (1 × 10^6^ cells) in the left fourth mammary gland fat pad to induce tumors. Mice were randomized once most tumors reached ~ 50 mm^3^ or greater and divided into two groups: the first (control) group received doxorubicin therapy (Sigma, D1515, St. Louis, MO, USA), and the second (test) group was continuously provided with both sodium nitrate (1 g·L^−1^) and sodium nitrite (0.9 g·L^−1^) in their drinking water (replaced weekly) in addition to the doxorubicin therapy, according to the schedule shown in Fig. 1. While nitrate supplementation (following the dosage used in the earlier acute Dox toxicity study by Zhu *et al*. [3]) can provide a long‐lasting, slow release of nitrite, we also supplied sodium nitrite directly in the drinking water (with dosage at an intermediate concentration relative to those used in Raat *et al*. [15]).

Thirteen days after tumor cell injection, 2.5 mg·kg^−1^ of intravenous doxorubicin was injected into the tail vein once weekly for 4 weeks to reach an accumulated dose of 10 mg·kg^−1^. Tumor volumes were measured twice per week and echocardiography was performed before the first and after the last Dox treatment. At the study endpoint 1 week after the final dose of Dox, mice were humanely euthanized by decapitation after isoflurane anesthesia and plasma, tumors, lungs, and heart were harvested for analysis. All protocols were approved by the Animal Care and Use Committee of the Wake Forest University School of Medicine (protocol #A17‐172, approved Feb 21, 2018), and all procedures were performed in accordance with relevant guidelines and regulations. Humane endpoints for mice followed the Standard Operating Procedures defined by the Wake Forest Institutional Animal Care and Use Committee Guide. In brief, animals would be euthanized at or before (a) the size or location of the neoplasm becomes a significant hindrance for eating, drinking or locomotion, (b) the neoplasm develops extensive and/or painful ulceration or necrosis; (c) signs of end‐stage illness are observed; or (d) the overall tumor burden is excessive (tumor burden greater than 10% body weight).

### Echocardiography

Transthoracic echocardiography was performed using a Vevo 2100 LAZR ultrasound system (FUJIFILM/VisualSonics, Inc.; Toronto, Canada) equipped with a 30 MHz linear array transducer. M‐mode short‐axis images were obtained to assess ejection fraction and fractional shortening.

### Nitrite and nitrate levels in plasma

Blood collected at the time of sacrifice was centrifuged (2500 ***g***) for 3 min at 4 °C, then plasma was aliquoted into tubes, placed on dry ice, and stored at −80 °C until analysis by chemiluminescence. Plasma nitrate (${\mathrm{NO}}_{3}^{-}$) and nitrite (${\mathrm{NO}}_{2}^{-}$) levels were measured using an HPLC‐based Eicom NOx Analyzer, model ENO‐20, according to the instructions of the manufacturer. For all measurements, standard curves were obtained and used for quantitative measurements.

### Tissue preparation and analysis

Tumors, lungs, and 2‐mm cross‐sectional slices taken approximately 5 mm from the bottom of the heart were fixed in 4% paraformaldehyde for 24 h before embedding in paraffin. Embedded lung and heart tissues were cut into 5‐μm‐thick sections and stained with hematoxylin and eosin (H&E). Lung metastatic lesions were quantified from the H&E images. Heart sections were also stained with PicroSirius Red for collagen, indicating areas of fibrosis; a total of 12 representative images from each group were quantified.

In addition, small pieces of cardiac tissue were rapidly frozen in liquid nitrogen at the time of sacrifice and used to conduct western blotting for 4‐hydroxynonenal (HNE) adducts on proteins, and for levels of peroxiredoxin‐3 (Prx3) and glyceraldehyde‐3‐phosphate dehydrogenase (GAPDH). Briefly, 11 mg of frozen heart tissue was weighed out and gently processed into a single‐cell suspension in 50 μL of sterile phosphate‐buffered saline using the soft end of the 1 mL syringe plunger, with samples stored at −20 °C until analysis. Levels of Prx3 (antibody from Abcam, ab73349, Cambridge, UK), HNE adducts (Abcam, ab46545), and GAPDH (EMD Millipore, CB1001, Burlington, MA, USA) were evaluated with three replicates per animal by loading 2.5 μL per sample on a 10% polyacrylamide gel for SDS/PAGE, followed by western blot analysis. Detection of antigens used horseradish peroxidase‐linked secondary antibodies (Anti‐rabbit IgG from Cell Signaling (Danvers, MA, USA), catalog # 7074 for HNE and Prx3 Western blots and Anti‐mouse IgG from Cell Signaling, catalog # 7076 for GAPDH) and SuperSignal West Dura Extended Duration Substrate kit from Thermo Scientific (Waltham, MA, USA). Images were collected with a KODAK imager and analyzed using the imagej software.

### Statistical analysis

Data are presented as the mean ± standard error of the mean (SEM). Statistical analysis of tumor volumes over time was conducted by two‐way analysis of variance (ANOVA); tumor volumes were obtained from measurements of the longest perpendicular axes ((long axes) × (short axes)^2^/2). Statistical analysis of other data was conducted using a nonpaired, two‐sided Student's *t*‐test (GraphPad Prism 10 Software, San Diego, CA, USA). The criterion for statistical significance was set at *P* < 0.05.

## Results

Based on the need to test nitrate/nitrite (i.e., NOx) effects on Dox cardiotoxicity and efficacy in tumor‐bearing animal models, we designed a focused study with Dox‐treated BALB/c mice bearing syngeneic breast cancer tumors where one of the two groups continuously received NOx in their drinking water; this mode of NOx administration is similar to the previous acute toxicity study with nontumor‐bearing male CF‐1 mice [3]. To maximize the ability to discern NOx effects, mice were maintained on a low nitrate diet [15]. As shown in Fig. 1A, syngeneic 4T1 TNBC cells were injected into one mammary fat pad per mouse (*N* = 10), then 1 week later, half of the mice (*N* = 5) were switched to the NOx‐containing water. Nitrate is the source of sustained NOx supplementation (through accumulation in the salivary glands and oral bioactivation); nitrite was also included as a more immediately bioavailable NOx source. Once tumors were palpable (about 2 weeks after injection of the 4T1 cells), echocardiography was performed, then all mice received four weekly intravenous doses of Dox (2.5 mg·kg^−1^ each). One week after the final dose of Dox, mice were again evaluated by echocardiography, then sacrificed to collect various tissues (including plasma, lung and heart tissue). Analyses of the plasma verified that the supplementation of the drinking water was effective in elevating plasma levels of both nitrite and nitrate (Fig. 1B,C and Table S1). Plasma analysis confirmed that the supplementation of the drinking water effectively elevated systemic NOx levels, as indicated by significantly increased plasma levels of both nitrite (1.48 μm) and nitrate (239 μm) (Fig. 1B,C and Table S1). These concentrations are in ranges readily achieved in human volunteers by drinking beet juice (e.g., 0.8–1.2 μm nitrite and 300–360 μm nitrate in several studies [16, 17]), and well below levels which can be achieved using infused nitrite (200 μm nitrite [18]).

**Fig. 2:**
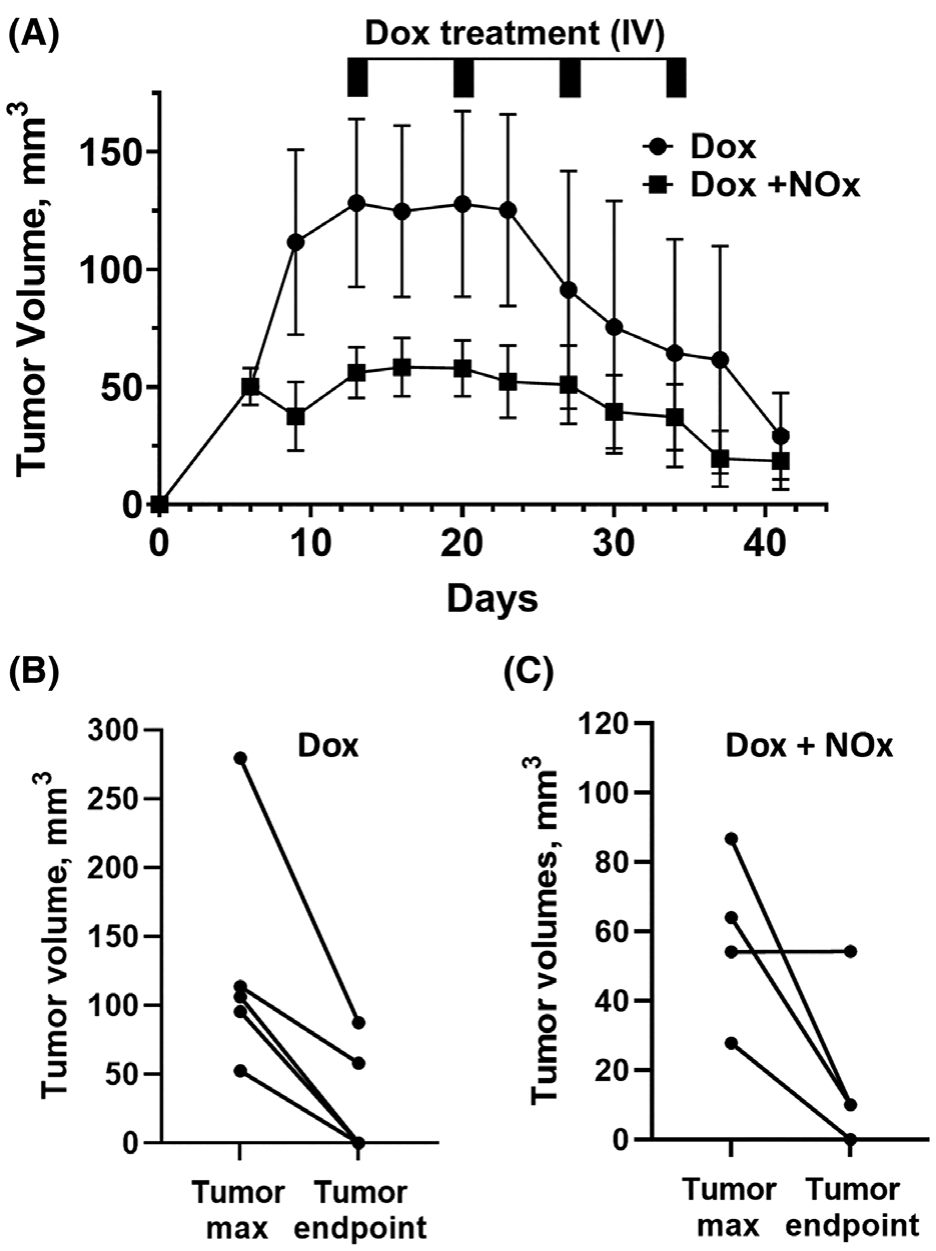
Tumor volumes of individual TNBC tumor‐bearing BALB/c mice during Dox treatment in the presence or absence of NOx (nitrate plus nitrite) supplementation. (A) Beginning 13 days after tumor cell injection, animals received four weekly intravenous (IV) doses of doxorubicin (2.5 mg·kg^−1^ each), and tumor size was measured twice weekly using calipers. Data are presented as the mean ± standard error of the mean (SEM), and statistical analysis was conducted by 2‐way analysis of variance (ANOVA). While the two groups of animals were not significantly different based on the row factor (time since inoculation), they were different based on treatment, with NOx‐supplemented animals overall presenting with smaller tumors (*P* = 0.0016), and there was no significant interaction between the two factors. (B and C) tumor volumes of individual mice treated with Dox alone (B), or Dox in combination with NOx‐supplemented H_2_O (C), were compared at their maximal size and at the endpoint of 41 days. One animal (which received NOx supplementation) lacked any tumor growth and was excluded from all data shown and used for statistical analysis in this and other figures. Therefore *n* = 5 for Dox alone, and *n* = 4 for the Dox + NOx group.

As shown in Fig. 2, tumor volumes among the 10 mice varied, with one mouse never developing a tumor and another developing an unusually large tumor. The mouse for which no tumor developed (in the NOx supplementation group) was removed from further statistical analysis here and below. Based on statistical analysis by two‐way analysis of variance (ANOVA), the two groups of animals were not significantly different based on the time since inoculation, but were different based on treatment, with NOx‐supplemented animals overall presenting with smaller tumors (*P* = 0.0016). Although the low animal numbers and variability limit the conclusions that can be made regarding any effect of NOx on Dox efficacy, our data suggest that dietary NOx supplementation does not impact Dox efficacy in this TNBC model.

**Fig. 3:**
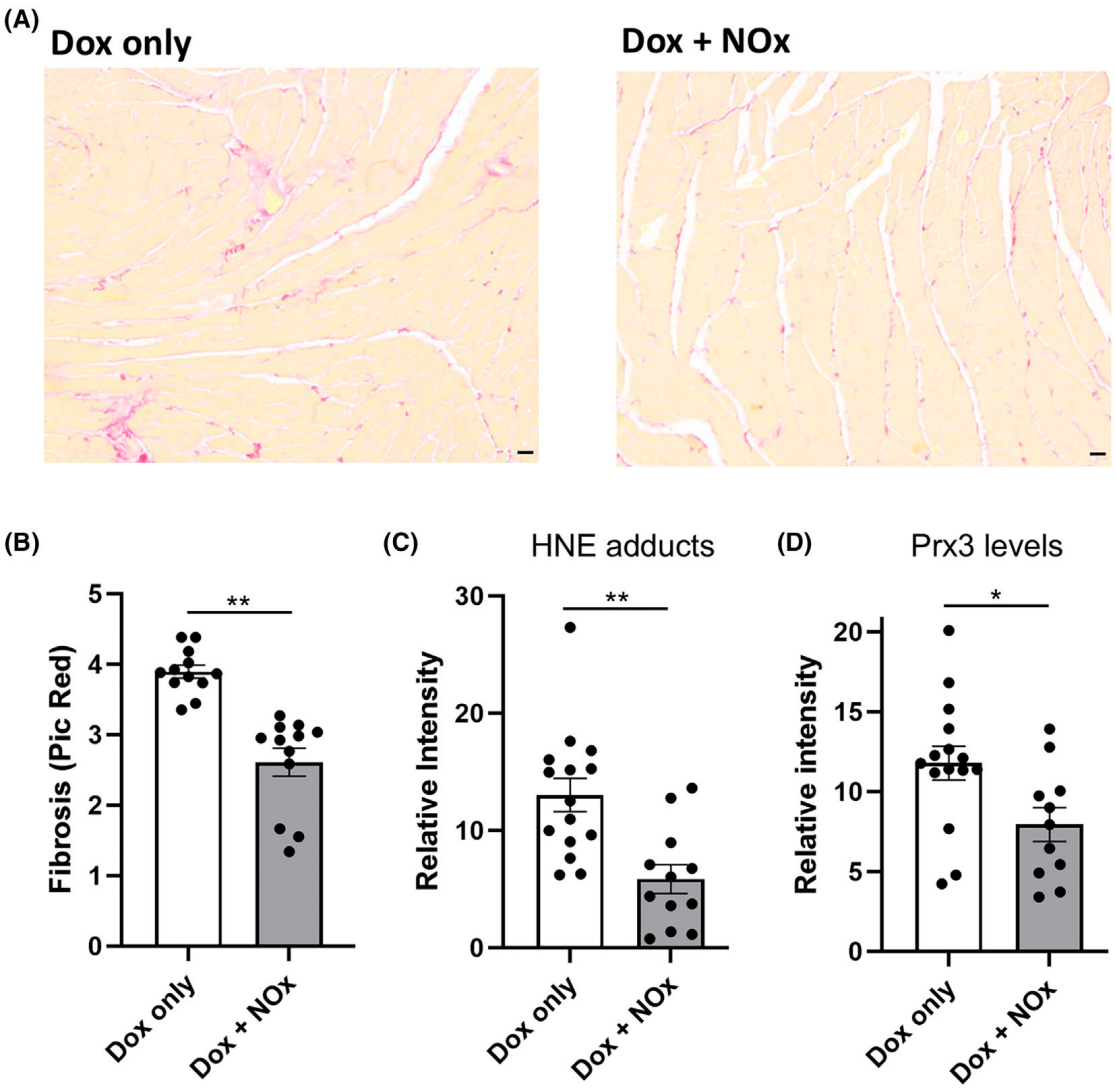
Protection of Dox‐treated cardiac tissue from fibrosis and formation of protein adducts with 4‐hydroxynonenal (HNE) by nitrate and nitrite supplementation. (A) Fibrosis was evaluated using PicroSirius Red staining of left ventricle (LV) cross‐sections. (B) Fibrosis was significantly decreased with NOx (nitrate plus nitrite) treatment. (C and D) HNE adducts (C) and Prx3 levels (D) were assessed by western blot analysis of frozen LV tissue, indicating statistically significant differences between groups. Data are presented as the mean ± standard error of the mean (SEM), and statistical analysis in (B) through (D) was conducted by two‐tailed Student's *t*‐test. As in Fig. 2, data from the non‐tumor‐bearing animal was excluded, so *n* = 5 and *n* = 4 (biological replicates) for Dox alone or with NOx supplementation, respectively. Multiple technical replicates of each are shown as each analysis was conducted on a separate piece of tissue. In (A) scale bars shown on images (lower right) are 100 μm. *, *P* < 0.05, **, *P* < 0.01.

While echocardiography data for both treatment groups did not reveal any Dox‐mediated deficits (and therefore also no rescue by NOx treatments) as assessed by ejection fraction (EF) or fractional shortening (FS) (Table 1), a significant decrease in Dox‐induced cardiac fibrosis assessed through PicroSirius Red staining of left ventricle (LV) slices was observed in the NOx‐treated animals (Fig. 3A,B). Moreover, oxidized lipid (4‐hydroxynonenal, HNE) adducts on proteins detected by Western blots of LV tissue were suppressed by NOx treatment (Fig. 3C). In addition, the mitochondrial antioxidant protein Prx3, shown previously to be increased after acute Dox treatment [12], was lower in the mice treated with NOx (Fig. 3D).

**Table 1:** Echocardiography results^a^.

Treatment, animal ID	Ejection fraction (%)		Fractional shortening (%)		Cardiac ouput (mL·min^−1^)		Heart rate (beats·min^−1^)		E/E'	
	Start	End	Start	End	Start	End	Start	End	Start	End
Dox only										
1751	64.3	61.0	34.6	32.0	16.2	15.5	385	446	41.4	44.7
1752	64.1	69.1	33.9	38.0	11.2	12.8	421	379	39.2	37.8
1753	61.0	61.0	31.7	31.8	11.7	11.1	406	417	30.4	43.2
1754	55.1	54.1	28.3	27.7	17.7	18.5	451	406	36.6	57.1
1755	66.5	65.1	36.0	35.0	17.9	18.8	327	486	36.8	48.0
Dox + NOx										
1756	54.8	64.6	28.0	34.6	15.5	17.8	409	402	31.9	56.4
1757	50.7	57.7	25.5	29.9	13.7	17.0	360	446	32.1	33.5
1758	68.5	59.2	37.6	30.7	13.5	13.4	345	390	39.0	52.1
1759	69.6	57.5	38.4	29.7	12.8	17.0	403	465	35.8	39.8
1760	57.1	59.0	29.7	30.7	19.2	15.6	377	484	42.7	47.9
Summary										
Dox only	62.2 ± 2.0	62.1 ± 2.5	32.9 ± 1.3	32.9 ± 1.7	14.9 ± 1.5	15.4 ± 1.5	398 ± 21	427 ± 18	36.9 ± 1.8	46.1 ± 3.2
Dox + NOx	60.1 ± 0.8	59.6 ± 1.3	31.8 ± 2.6	31.1 ± 0.9	15.0 ± 1.1	16.2 ± 0.8	379 ± 12	437 ± 18	36.3 ± 2.1	45.9 ± 4.1

^a^ Transthoracic echocardiography was performed using a Vevo 2100 LAZR ultrasound system (FUJIFILM/VisualSonics, Inc.; Toronto, Canada) equipped with a 30 MHz linear array transducer. M‐mode short‐axis images were obtained to assess ejection fraction and fractional shortening. The ‘Start’ data were collected after tumor growth and supplementation of the Dox + NOx group with nitrate and nitrite in their drinking water according to the experimental plan outlined in Fig. 1A. ‘End’ data were taken approximately 1 week after the last Dox treatment, prior to sacrifice.

**Fig. 4:**
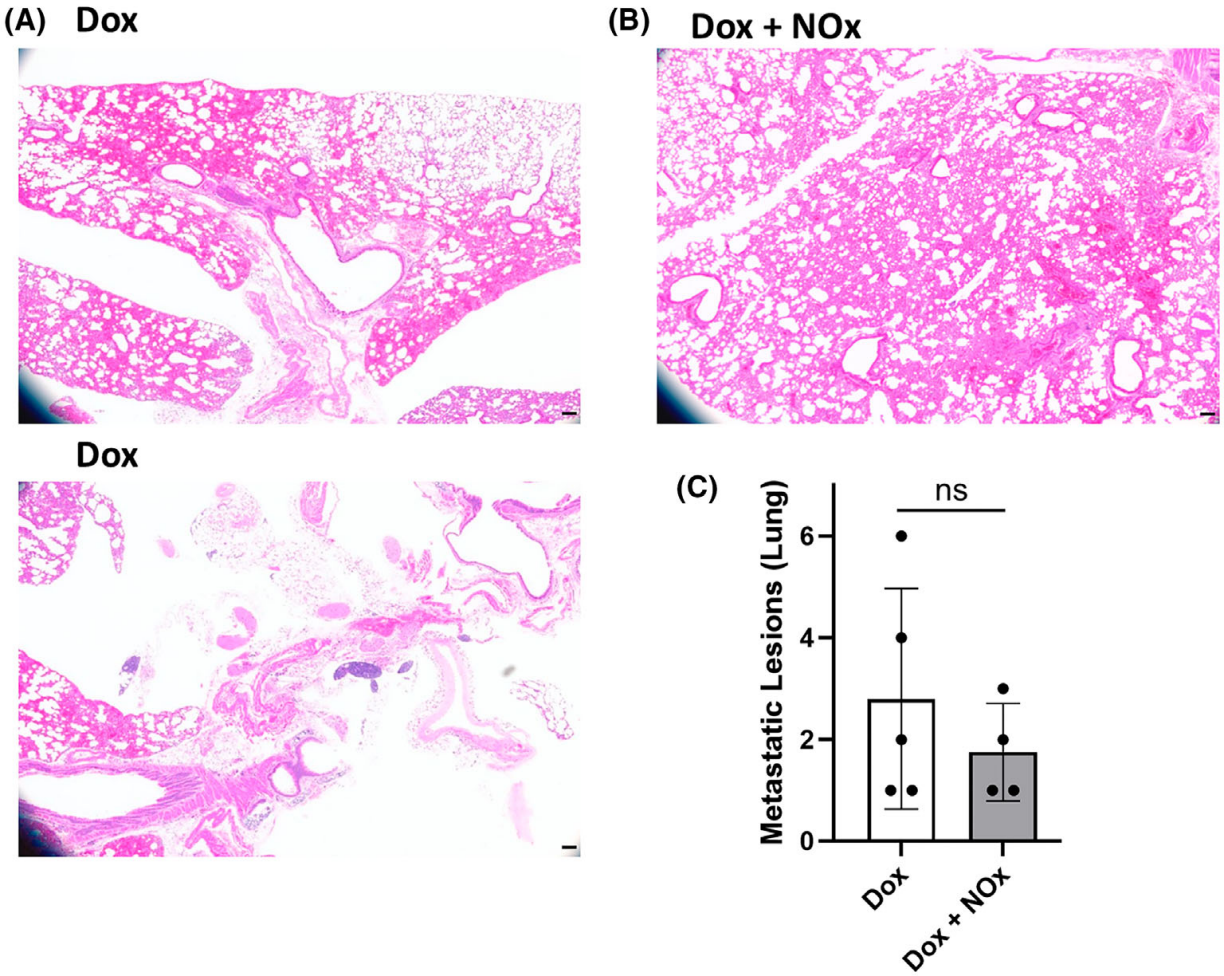
Effects of NOx (nitrate plus nitrite) supplementation on lung tissues and TNBC‐associated metastatic lesions. (A and B) Hematoxylin and Eosin (H&E) stained lung tissue from Dox‐exposed mice showed significant evidence of tissue remodeling (A), which was less evident in the tissue from the NOx‐supplemented mice (B). (C) Numbers of metastatic lesions in lung tissue associated with TNBC and Dox treatment were similar but trended lower in the NOx‐supplemented animals (*P* = 0.237). As in the other figures, data from the non‐tumor‐bearing animal was excluded, so *n* = 5 and *n* = 4 for Dox alone or with NOx supplementation, respectively. For (A) and (B), scale bars shown on images (lower right) are 100 μm. Statistical analysis in (C) was conducted by two‐tailed Student's *t*‐test. ns, not significant.

Finally, metastatic lesions in lung tissue were quantified from slices of formalin‐fixed lung tissue stained with Hematoxylin and Eosin. The lung slices were visually distinct between the NOx‐treated and untreated animals, showing significant pathology in the absence of NOx (Fig. 4A,B). Quantitation of metastatic lesions suggested fewer metastatic lesions in the NOx‐treated mice (Fig. 4C). Still, this difference was not statistically significant (in part due to the overall low numbers of lesions as well as limited animal numbers). Importantly, there was no evidence that NOx supplementation caused an increase in lung metastases, which would be an outcome of concern.

## Discussion

Multiple cardioprotective therapies are under investigation through preclinical or early clinical trials. Still, clinically approved treatments to address cardiotoxicity of chemotherapeutics, other than dexrazoxane, which is only approved for limited use, are not yet available. Inorganic nitrate (for example using beet root juice supplementation) holds promise as a cardioprotective agent given its demonstrated ability, in an acute Dox‐exposure animal model, to preserve cardiac function and protect from oxidative damage [3]. In light of those findings in the absence of cancer, our team set out to determine whether the cardioprotective effect of nitrate would extend to TNBC tumor‐bearing mice undergoing Dox‐mediated therapy. We also sought preliminary indications of any potential adverse effects with respect to limitations on Dox efficacy or enhanced metastases to lung with nitrate supplementation, although a larger study will be needed to better address these issues. To ensure both short‐term and long‐term availability of NOx species, both ${\mathrm{NO}}_{2}^{-}$ and ${\mathrm{NO}}_{3}^{-}$ were included in the drinking water. While one animal in the NOx‐treated group did not develop a tumor and tumor volumes were variable in the other mice, there was no indication that NOx supplementation impacted Dox antitumor activity (Fig. 2A–C). Analysis of the harvested tissues clearly indicated the protection, by NOx supplementation, of cardiac tissue from Dox‐induced fibrosis (Fig. 3A,B) and oxidation‐related protein damage as shown by HNE adducts detected by western blot (Fig. 3C). Prx3 levels, which are known to rise with Dox treatment [12, 13], were also lower (Fig. 3D), perhaps due to the lower ROS levels in the NOx‐protected animals. Functional studies using echocardiography (Table 1) were uninformative as there was no difference in EF or FS values with either Dox alone or in combination with NOx. It appears that protection of cardiac function by oral nitrate demonstrated previously in male CF‐1 mice acutely exposed to Dox [3] was not detectable here in part because our chronic exposure model of breast cancer tumor‐bearing female BALB/c mice exhibited lower EF and FS values at the outset that were not affected by Dox at the timepoints assessed (Table 1). Finally, and importantly, there was no evidence for an enhancement in metastatic TNBC lesions in the lungs of those animals supplemented with NOx; if anything, there was a trend toward fewer metastatic lesions in the NOx‐treated animals. While preliminary, this study supports the need to study dietary nitrate and nitrite more deeply as cardioprotective agents compatible with anthracycline treatment. We postulate that nitrate, with or without added nitrite, may even lower the effective dose of Dox needed to treat the cancer; if true, this would further lower the risk of cardiovascular damage by anthracycline treatment. With its cardioprotective effects demonstrated here in a tumor‐bearing mouse model, inorganic nitrate as an oral supplement could provide a cheap and convenient way to lower the risk of adverse cardiovascular effects of anthracycline treatment (as long as further confirmation is obtained that the anticancer effects of Dox are not impaired and the risk of metastatic spread of the cancer does not increase). Moreover, pre‐existing hypertension in some patients exacerbates the cardiotoxic effects of chemotherapies, and as shown for pulmonary arterial hypertension noted above [11], NOx could also offer particular benefits for these patients.

It is well‐established that ROS generation plays a role in Dox cardiotoxicity, with involvement of (a) mitochondria‐dependent production of ROS (in part due to accumulation of Dox and its binding to cardiolipin and redox cycling to produce superoxide in this organelle), (b) ROS production from uncoupled (damaged) endothelial nitric oxide synthase (eNOS), and (c) exacerbation of Dox cytotoxicity when cellular free iron levels are increased [2, 19, 20]. As yet, administration of simple antioxidants such as Vitamin E have not proven to be particularly effective, although other therapies continue to be explored for which lowering ROS is likely to contribute to the preliminary cardioprotective effects observed (e.g., with ACE inhibitors as well as NLRP3 inflammasome inhibitors like the flavonoid dihydromyricetin and resveratrol) [20, 21]. Dexrazoxane, initially utilized to counteract Dox toxicity based on its iron‐chelating activity, was subsequently demonstrated to exert its cardioprotective effect primarily through its depletion of Topo2B, thereby limiting DNA damage and inflammatory responses [2]. It should be noted that ROS and reactive nitrogen species (RNS), and especially their interactions, are complex and highly dependent on context as their pleotropic effects, sometimes protective and sometimes damaging, are dependent on fluxes through multiple competing pathways (for example, accelerating superoxide removal by superoxide dismutase activity may suppress its reaction with nitric oxide to form peroxynitrite, but also enhances the production of hydrogen peroxide, another form of ROS). It is also important to recognize that simple chemical nitric oxide donors utilized in cell culture or animals do not exhibit the same properties as NOx *in vivo* [19], thus preclinical and clinical testing is imperative to test the effects of dietary NOx administration properly. Indeed, as nitrite forms ^·^NO preferentially in areas of low oxygen, it differs from other ^·^NO‐producing agents, a feature that could be particularly important for cardioprotective effects against chemotherapies or hypoxia/reperfusion injury.

There is an expanding appreciation for the nitrate–nitrite–NO axis as an alternative way to generate protective ^·^NO, particularly as nitric oxide synthases cannot function without oxygen, which is a substrate. There is also a building recognition that the hypoxia‐induced release of ^·^NO from bioactivated nitrate in blood and other tissues is necessary for matching oxygen delivery to demand [10, 22]. Bioactivation of nitrite occurs through nitrite reductase activity of enzymes such as xanthine oxidoreductase and heme‐containing proteins such as hemoglobin and myoglobin [23, 24, 25], with the latter shown to be critical to the protection from I/R injury in isolated hearts and mouse models [24]. Based on our work and that of others, it seems prudent to further pursue dietary inorganic nitrate as a protective agent against the insipid cardiotoxicity associated in many patients with chemotherapeutic treatments that effectively treat cancers but create a large survivor population with a potentially avoidable, long‐persisting elevation in risk of cardiac failure.

## Conflict of interest

The sponsors had no role in the design of the study, in the collection, analyses, or interpretation of data; in the writing of the manuscript; or in the decision to publish the results. DBK‐S is co‐inventor on patents directed to the use of nitrite salts in cardiovascular diseases, which were previously licensed to United Therapeutics, and licensed to Globin Solutions and Hope Pharmaceuticals. DBK‐S is inventor on a patent filed through the University of Pittsburgh related to the creation and use of NO‐ferroheme. DBK‐S owns stock in and serves on the scientific advisory board for Beverage Operations LLC, which has licensed Wake Forest University intellectual properties and thus has a financial interest in Beverage Operations LLC (which has sold beet juice). Other authors declare no conflict of interest.

## Author contributions

Conceptualization, LBP; experimental data collection, RDY, XC, NC‐D, SB; data analysis, RDY, DBK‐S, DRS‐P, LBP; writing – original, review, and editing, RDY, XZ, DBK‐S, DRS‐P and LBP. All authors have read and approved the final manuscript.

## Supporting information


**Fig. S1.** Representative Western blots of cardiac samples detecting HNE adducts, GAPDH and Prx3 proteins used in quantitation for Fig. 3C,D.
**Table S1.** Nitrate and nitrite levels in the plasma of NOx‐treated and untreated mice.

## Data Availability

The data that support the findings of this study are available in the Supporting Information linked to this manuscript and as openly shared data in Dryad at: https://doi.org/10.5061/dryad.ghx3ffc1p. The spreadsheets provide the following detailed information:
NOx (nitrate and nitrite) levels in plasma, μm.
Tumor volumes, each animal, mm^3^. Maximum and endpoint tumor volumes, each animal, mm^3^. Fibrosis data based on PicroSirius Red staining of cardiac tissue slices. 4‐Hydroxynonenal adduct formation on proteins (western blot densities) of cardiac tissue samples. Prx3 levels (western blot densities) of cardiac tissue samples. Numbers of metastatic lesions in lung tissue slices. Echocardiography data of all animals before and after Dox treatments.
