# Supplementary material for: Dietary nitrate and nitrite protect against doxorubicin‐induced cardiac fibrosis and oxidative protein damage in tumor‐bearing mice
Source: FEBS Open Bio. 2025 Nov 20;16(3):584–94. doi: 10.1002/2211-5463.70139 (PMC12955748; doi:10.1002/2211-5463.70139)
Supplement: Supplementary file 1 — Fig. S1. Representative Western blots of cardiac samples detecting HNE adducts, GAPDH and Prx3 proteins used in quantitation for Fig. 3C,D. Table S1. Nitrate and nitrite levels in the plasma of NOx‐treated and untreated mice. [file FEB4-16-584-s001.pdf]

Dietary nitrate and nitrite protect against doxorubicin-induced cardiac fibrosis and oxidative protein damage in tumor-bearing mice

Rama D. Yammani, Xiaofei Chen, Nildris Cruz-Diaz, Xuewei Zhu, Swati Basu, Daniel B. Kim-Shapiro, David R. Soto-Pantoja, and Leslie B. Poole

Supporting Information

Detailed information about the plasma NOx concentrations are provided in Table S1. Figure S1 includes representative Western blots used for quantitation shown in Figures 3C and 3D.

Table S1. Nitrate and nitrite levels in the plasma of NOx-treated and untreated mice.

NOx species measured (μM)	Group 1 (Dox)	Group 2 (Dox with NOx)
Nitrite	0.106 (± 0.008)	1.48 (± 0.49)
Nitrate	12.2 (± 0.9)	239 (± 57)

Blood samples taken at the time of sacrifice were rapidly centrifuged at 4 °C and stored at -80 °C until analysis by chemiluminescence as described in the Methods. Shown are the mean values ± standard error.

Figure S1. Representative Western blots of cardiac samples detecting HNE adducts, GAPDH and Prx3 proteins used in quantitation for Figures 3C and 3D.

A

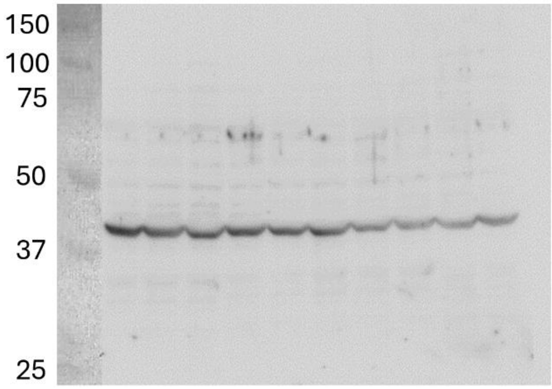

B

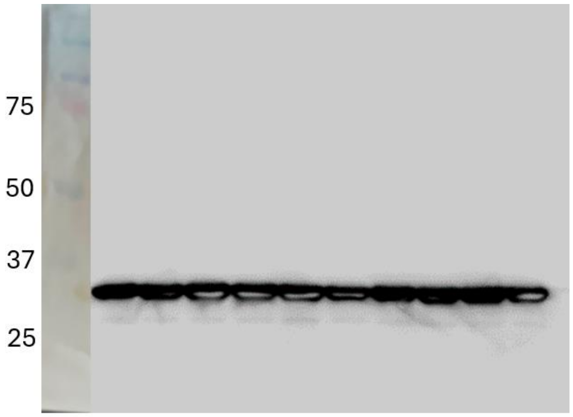

C

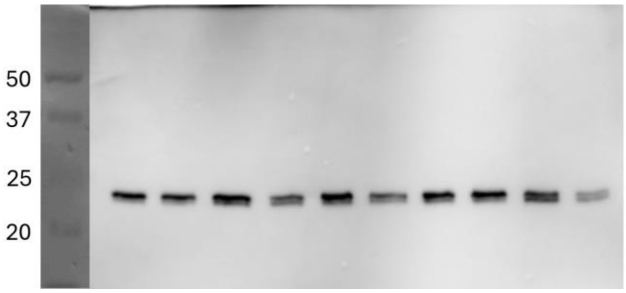

The blots were performed using samples of frozen heart tissue prepared and analyzed on a 10% SDS-polyacrylamide gel under reducing conditions followed by electrotransfer and detection using individual antibodies against each target, as described in the Methods. Analytes were detected using the SuperSignal West Dura Extended Duration Substrate kit from Thermo Scientific, and images were collected with a KODAK imager and analyzed using ImageJ software. Separate images (spliced in to the left of each blot) allowing the molecular weight standards to be seen have been aligned with each gel and marked (in kDa). **A**, representative blot for HNE adducts on proteins from cardiac tissue of all study mice. **B** and **C**, representative blots for GAPDH (as a loading control) and Prx3, respectively, analyzing cardiac tissue of all study mice. While HNE is present on multiple proteins (A), expected molecular weights for GAPDH (B) and Prx3 (C, also known as Prdx3) are 35.8 kDa and 21.7 kDa, respectively. For A-C, samples from all five animals from each group were analyzed simultaneously, with the left 5 samples taken from animals receiving no supplementation in their water and the right 5 samples taken from animals receiving NOx supplementation.
